# Supplementary material for: Nonlinear spin-orbit coupling in optical thin films
Source: Nat Commun. 2024 Feb 22;15:1625. doi: 10.1038/s41467-024-45607-2 (PMC10884006; doi:10.1038/s41467-024-45607-2)
Supplement: Supplementary file 1 — Supplementary Information [file 41467_2024_45607_MOESM1_ESM.pdf]

Supplementary Information for

Nonlinear spin-orbit coupling in optical thin films

Domenico de Ceglia[1,2*], Laure Coudrat[3*], Iännis Roland[3], Maria Antonietta Vincenti[1,2], Michael Scalora[4], Rana Tanos[5], Julien Claudon[5], Jean-Michel Gérard[5], Aloyse Degiron[3], Giuseppe Leo[3,6] and Costantino De Angelis[1,2]

[1]CNIT and Department of Information Engineering, University of Brescia, Via Branze, 38, Brescia, 25123, Italy.
[2]Istituto Nazionale di Ottica, Consiglio Nazionale delle Ricerche, Via Branze, 45, Brescia, 25123, Italy.
[3]Université Paris Cité, CNRS, Laboratoire Matériaux et Phénomènes Quantiques, 10 rue A. Domon et L. Duquet, Paris, 75013, France.
[4]Charles M. Bowden Research Center, Redstone Arsenal, 35898-5000, USA.
[5]Univ. Grenoble Alpes, CEA, Grenoble INP, IRIG, PHELIQS, "Nanophysique et Semiconducteurs" Group, Grenoble, F-38000, France.
[6]Institut universitaire de France (IUF).

*Corresponding authors. E-mail: domenico.deceglia@unibs.it, laure.coudrat@u-paris.fr

The PDF file includes:

SUPPLEMENTARY FIGURES 2
Supplementary Figure 1: Experimental setup 2
Supplementary Figure 2: Characterization of the spectral properties of the SH signal 3
Supplementary Figure 3: Measurement of time averaged SH vs FF power 4
Supplementary Figure 4: Polarization state of the SH 5

SUPPLEMENTARY FIGURES

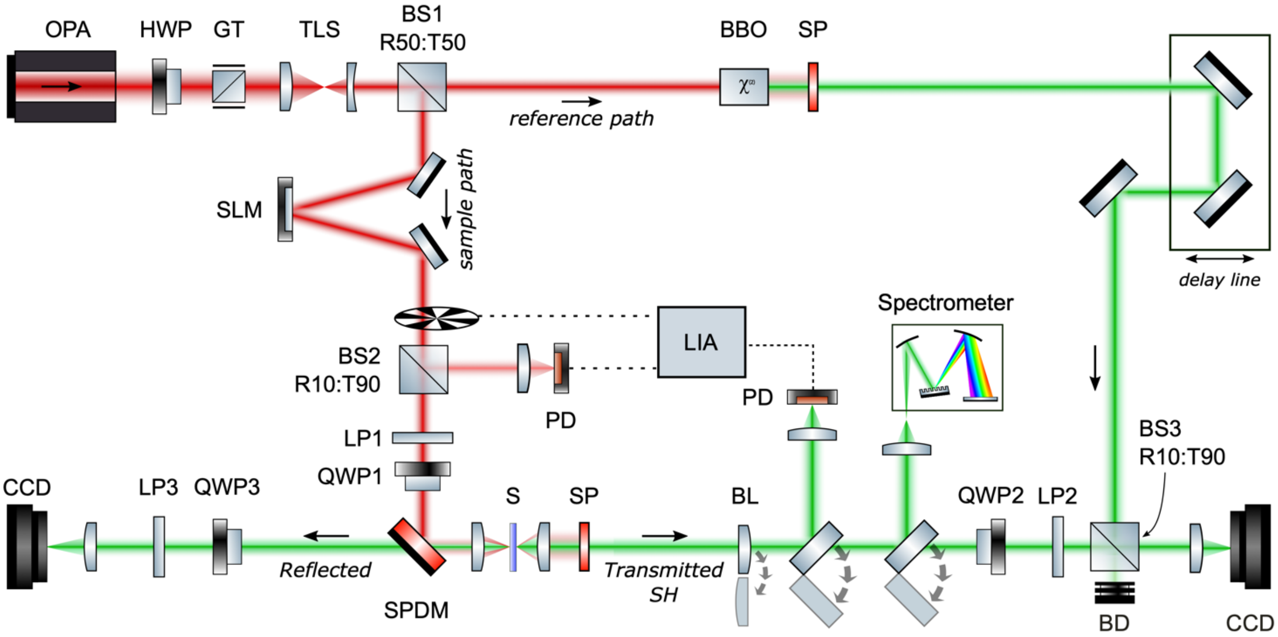

Supplementary Figure 1: Experimental setup

Experimental setup for SH characterization. Pump (red) and SH (green) beams, optical parametric amplifier (OPA), half-wave plate (HWP), Glan Taylor polarizer (GT), telescope (TLS), beam splitter (BS), spatial light modulator (SLM), linear polarizer (LP), quarter-wave plate (QWP), short-pass dichroic mirror (SPDM), AlGaAs sample (S), short-pass filter (SP), Bertrand lens (BL), lock-in-amplifier (LIA), photodiode (PD), beam dump (BD), charge coupled device (CCD).

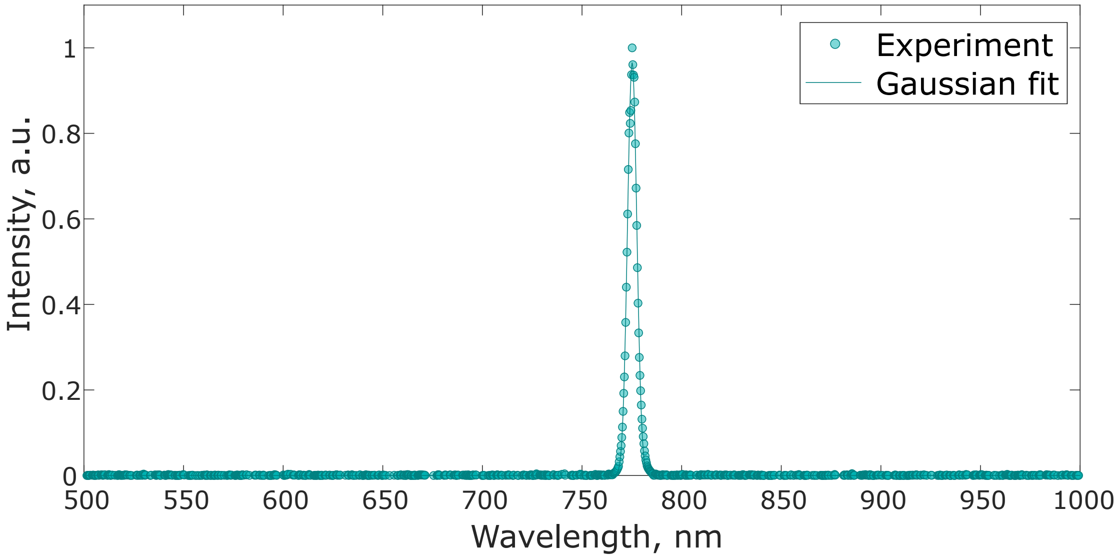

Supplementary Figure 2: Characterization of the spectral properties of the SH signal

SH spectrum of a (001) AlGaAs membrane for a 1550 nm pump wavelength.

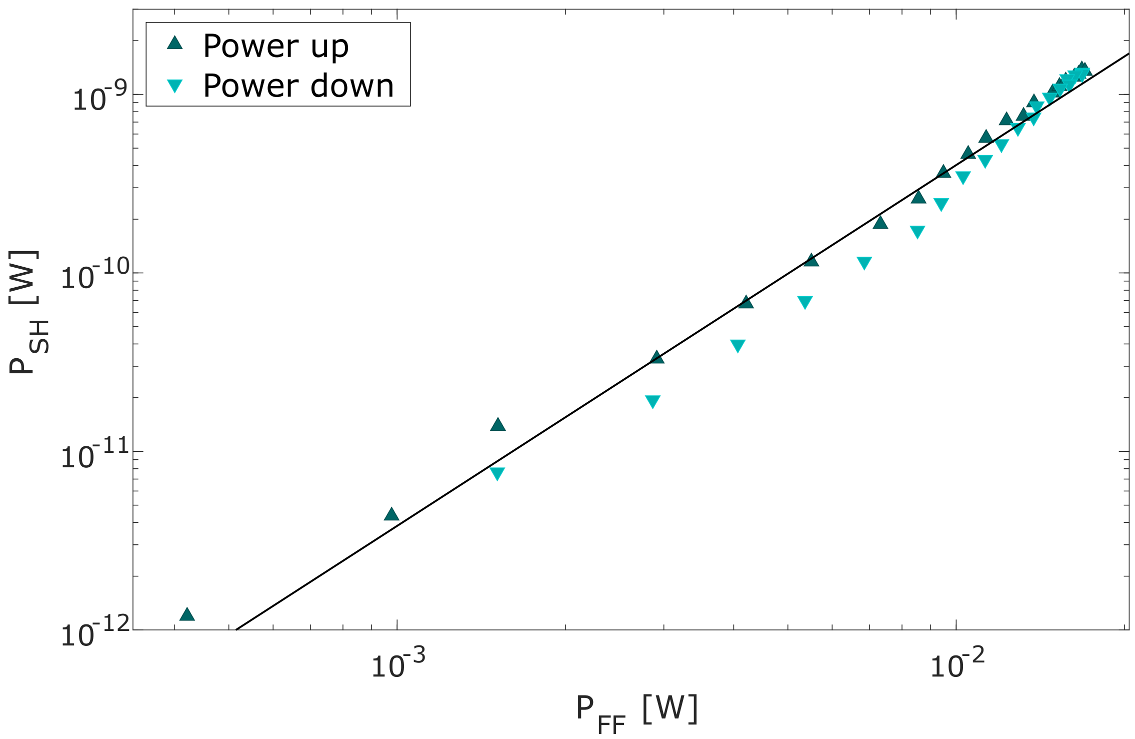

Supplementary Figure 3: Measurement of time averaged SH vs FF power

Triangles pointing up (down) refer to increasing (decreasing) power. The sample does not undergo irreversible processes.

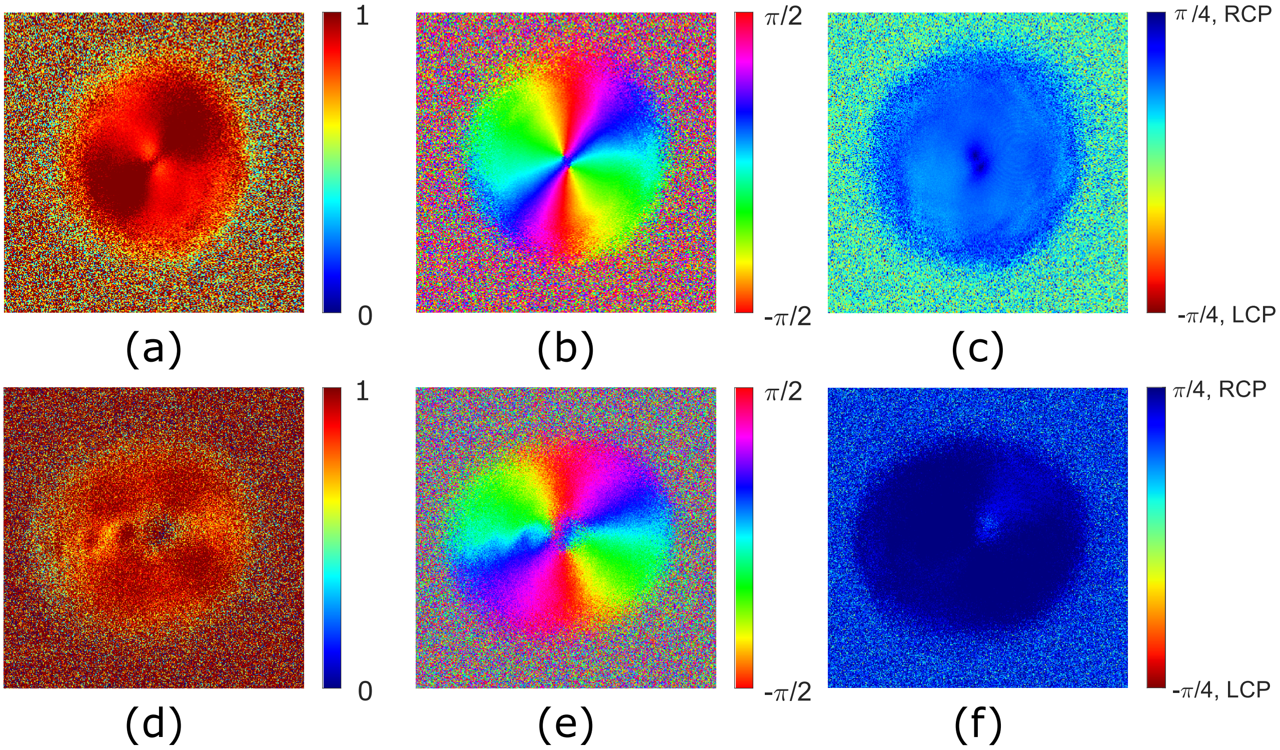

Supplementary Figure 4: Polarization state of the SH

Measuring the polarization state of the SH beams presented in Fig.2c (top row) and Fig.2d (bottom row). (a),(d) Degree of polarization p. (b),(e) Inclination angle ψ and (c),(f) ellipticity angle χ of the polarization ellipse.
